# Multiple Cranial Nerve Palsies as the First Presentation of Sarcoidosis

**DOI:** 10.1155/2014/592510

**Published:** 2014-03-26

**Authors:** Oliver Rose, Zahoor Ahmad, Barry Snow

**Affiliations:** ^1^Auckland City Hospital, Auckland, New Zealand; ^2^Manukau Superclinic, Auckland, New Zealand

## Abstract

Sarcoidosis is a disease process which predominantly affects the lungs but can involve virtually any organ in the human body. Neurosarcoidosis is a rare manifestation which can present in a variety of ways. There is no single diagnostic test for sarcoidosis; hence, the diagnosis is based on combined clinical, laboratorial, and radiological grounds. We describe a rare case where a patient presented with dysphagia, hoarseness, hearing loss, and unsteadiness.

## 1. Introduction

Sarcoidosis is a multisystem granulomatous disorder of unknown cause. Although it can involve virtually any organ system, it shows a predilection for the lungs with up to 90% of cases displaying pulmonary sarcoidosis. This is followed by ocular, skin, lymph node, and salivary gland involvement. Neurosarcoidosis is relatively rare accounting for approximately 5–15% of cases [1, 2]. As the disease can present in a variety of ways and to multiple medical specialities, it can be a challenge to diagnose and treat promptly with the potential for catastrophic complications. Cranial neuropathy appears to be the most frequent presentation of Neurosarcoidosis with the 7th cranial nerve being the most frequently affected nerve followed by the optic nerve [1, 2]. A presentation with multiple cranial nerve palsies is rare, and, hence, we present a case report with the involvement of cranial nerves V, VII, VIII, IX, and X.

## 2. Case

A previously healthy 32-year-old lady presented acutely to the ORL service with a 2-day history of increasing dysphagia, hoarseness of voice, and a blocked sensation in the right ear. She was unable to swallow any solids and experienced choking spells with thin fluids. She also described a recent viral-like illness that lasted for about 2 weeks followed by an episode of left sided facial weakness and asymmetry earlier during the week which seemed to have resolved completely at the time of presentation. On further questioning, she reported vague symptoms of unsteadiness especially while driving her car.

On examination, she had a very husky voice associated with difficulty in swallowing her saliva. She had weak palatal muscles and an absent gag reflex. Flexible laryngoscopic examination showed marked hypomobility of the left vocal cord and pooling of secretions in the hypopharynx. Pure tone audiogram showed a mild, high frequency sensorineural hearing loss on the left and a downsloping mild to moderate sensorineural hearing loss on the right side, (as illustrated in Figure 2). She had bilateral supraclavicular lymphadenopathy. Cerebellar and vestibular testing was unremarkable. There was no associated stridor or fever. Routine blood tests including an autoimmune screen were unremarkable. Brain MRI showed subtle findings of enhancement localised to the cisternal portions of the right and left VII and VIII cranial nerve complexes and also to the preganglionic portion of the right cranial nerve V. A CT scan demonstrated mediastinal, supraclavicular, and retroperitoneal lymphadenopathy.

Supraclavicular lymph node core biopsy showed granulomatous lymphadenitis. She was diagnosed with sarcoidosis on the grounds of clinical, radiological, and histological findings.

She made a full recovery after a course of corticosteroids.

## 3. Discussion and Literature Review

The diagnosis of neurosarcoidosis can be difficult to make due to the variability of presentation which includes cranial nerve palsies, meningitis, seizures, and neuropsychiatric symptoms [1, 2].

Multiple cranial neuropathies are uncommon presentations with diverse causes. Tumours in general appear to be the commonest cause accounting for about 30% of cases. This is followed by vascular disease, trauma, and infection. In Keane's 34-year review of 979 cases of multiple cranial palsies, sarcoidosis was the cause in 2 patients only [3].

The most common neurologic manifestation of sarcoidosis is cranial neuropathy secondary to nerve granulomas, raised intracranial pressure, or granulomatous meningitis [2]. The facial nerve is the most frequently affected cranial nerve. The paralysis is usually temporary and unilateral but it can also be bilateral in a simultaneous or sequential manner. The optic nerve appears to be the second most commonly involved cranial nerve [1, 2, 4]. Patients usually present with blurred vision or visual field defects. Multiple sclerosis is an important diagnosis to consider in cases of optic neuritis. Bilateral optic neuritis has a very poor prognosis in terms of recovery of reduced vision when compared with unilateral disease [2, 4]. Other cranial nerves are less frequently affected; the vestibularcochlear nerve is involved in 1–7% of cases of neurosarcoidosis. The hearing loss is usually sudden or rapidly progressive, bilateral, asymmetrical, and sensorineural in nature. Hearing loss is commonly associated with of vestibular impairment. In many cases, recovery of hearing loss is to be expected especially when there is radiological evidence of an enhancing retrocochlear lesion likely representing inflammation that would be responsive to corticosteroids [5].

**Figure 1:**
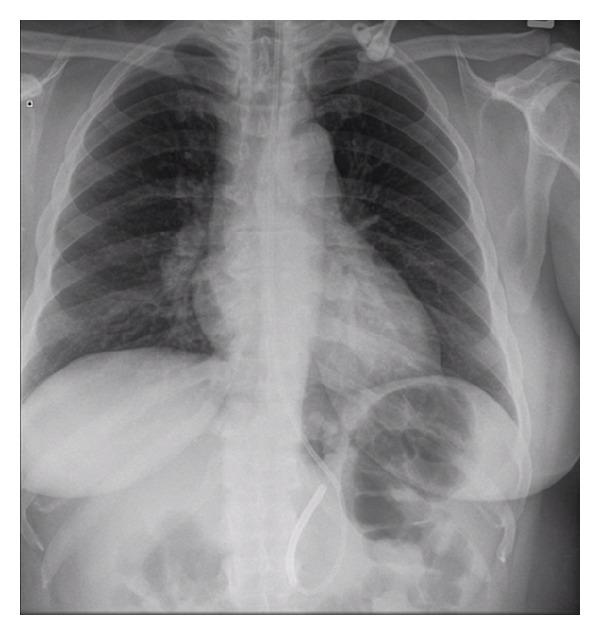
Chest X-ray showing hilar lymphadenopathy. Also of note is a nasogastric tube that was required for nutrition due to difficulty with swallowing.

**Figure 2:**
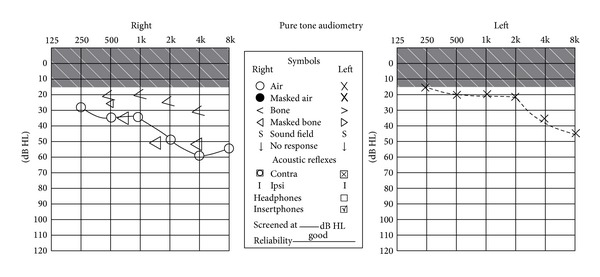
Pure tone audiogram showing mild, high frequency sensorineural hearing loss on the left and downsloping mild to moderate sensorineural hearing loss on the right side.

Although the cause of sarcoidosis is unknown, an abnormal immune response to infectious and noninfectious environmental factors appears to be a key factor [1, 2]. This theory may account for the prodromal symptoms experienced by our patient. The diagnosis is based on clinical and radiological correlation in combination with a tissue biopsy [1, 2]. Although commonly requested, serum angiotensin converting enzyme (ACE) has a poor predictive value in sarcoidosis. In our patient the serum ACE level was at the upper limit of normal. Furthermore, ACE can also be elevated in several disorders such as tuberculosis, multiple sclerosis, and Guillain-Barre Syndrome. MRI is sensitive for detecting CNS lesions of which leptomeningeal involvement with contrast enhancement is the most common finding; however, their appearances are highly variable, hence, reducing the specificity. Other findings include isolated mass lesions and diffuse inflammatory changes [1, 2]. A normal MRI does not rule out the possibility of neurosarcoidosis [1]. A chest X-ray is helpful, as it can display hilar lymphadenopathy as was the case in our patient, (as shown in Figure 1).

There are various diagnostic criteria suggested for neurosarcoidosis, and these classify cases as definite, probable, and possible neurosarcoidosis. A CNS or extraneural biopsy is usually required for a diagnosis of definite sarcoidosis [1].

Although there are no established treatment guidelines in neurosarcoidosis, corticosteroids are the first drugs of choice and patients usually respond to high doses. Cytotoxic drugs such as methotrexate and azathioprine can be used in patients where corticosteroids are contraindicated or in cases that are resistant to corticosteroids. Surgical intervention is reserved from life-threatening situations with large intracranial or spinal granulomas [1, 2].

Neurosarcoidosis is still a diagnostic dilemma due to its rarity and variable presentation. However, cranial nerve palsies are common presentations in otolaryngology; hence, granulomatous disorders such as sarcoidosis should always be part of the differential diagnosis.
